# A study on users’ preference towards diabetes-related video clips on YouTube

**DOI:** 10.1186/s12911-020-1035-1

**Published:** 2020-02-28

**Authors:** Jin Zhang, Zhong Zheng, Yanyan Wang, Yifan Zhu

**Affiliations:** 1 School of Information Studies, University of Wisconsin Milwaukee, Milwaukee, WI USA; 2 School of Information Management, Sun Yat-sen University, Guangzhou, People’s Republic of China; 3 School of Information Resource Management, Renmin University of China, Beijing, People’s Republic of China

**Keywords:** Video-based social media, YouTube, Content evaluation, Diabetes, User behavior

## Abstract

**Background:**

Social media has arisen to be a new and important channel for information users for seeking and creating user-generated content. For health consumers, social media has long been regarded and employed as an important source to find health-related information and emotional support. This study investigated the characteristics of diabetes-related videos posted on YouTube, one of the most popular video-based social media platforms, and explored the factors influencing users’ preference towards the investigated videos.

**Methods:**

A mixed research method including coding and negative binomial regression test was applied. Coding was utilized to identify the status of the diabetes-related video clips and the factors related to users’ attitude to them. Negative binomial regression approach was employed to detect significant relationships among the factors and users’ attitude.

**Results:**

The researchers selected eight factors (e.g. number of views, post period, presenters’ gender, and subject) to represent the characteristics of the diabetes-related video clips. Eleven subjects were identified by examining the diabetes-related videos and three subjects, Treatment, Sign & Symptom, and Social & Culture, appeared the most frequently. Media type, presentation setting, post period, presenter role, and presenters’ gender affect the users’ positive attitude significantly. Post period, presenter role, and the Sign & Symptom subject and the Nutrient subject have significant influence on the users’ negative attitude.

**Conclusions:**

Treatment, Sign & Symptom, and Social & Culture are the most popular subjects of the investigated video clips. The users are less likely to show their attitude to old videos. They prefer journalists and patients on videos but dislike male presenters compared with other presenters, and show more negative attitude towards the videos about nutrients. The findings of this study can be used to enhance the content creation of diabetes-related video clips for video contributors, the design and organization of the diabetes-related content for multimedia-based social media Website designers, and the information seeking and communication among health information users.

## Background

Social media has arisen to be a new and important channel for information users for seeking and creating user-generated content. As a powerful tool, social media provides patients and health professionals with a new channel to communicate and collaborate regarding health issues [1]. Because of its “instant and borderless”, the old monologue with one authority and multiple health consumers has been replaced by the many-to-many and close face-to-face dialogue where every patient with Internet access is able to become a content maker or receiver [2].

While a picture has long been considered to be worth more than a thousand words, multimedia-related information technology such as information visualization and graphical interfaces had not obtained an expanding role until the ability of computers to display resolution increased greatly in recent decades [3]. With mature information communication technology, streaming a video has also become ordinary in daily lives, which provides individuals with chances to select various forms of information that fit their needs best. In health care area, some people have used social media mainly based on textual information like Facebook and Twitter to exchange clinical information, medical guidance, professional feedback, and emotional support [4], while others have utilized Websites mainly based on multimedia information like YouTube to share symptoms, diagnosis, medication, and even personal stories [1].

As firmly established social media Website, YouTube has obtained popularity among health consumers and providers both in the United States and Europe, and approximately 100 million people have been reported to take some kinds of social interaction on YouTube [5]. Therefore, this study selected YouTube as the social media platform to further learn about information behavior among health consumers. However, studies also argue that information on Websites like YouTube might not be supervised, thus simply not being helpful for users who are seeking for assistance [5]. This concern could be reflected by certain user features such as “dislike” [6]. In the meanwhile, the responsibility of health professionals turns into ensuring the information offered is accessible and accurate [7].

According to the definition claimed by the 2014 National Diabetes Statistics Report [8], “Diabetes is a group of diseases marked by high levels of blood glucose resulting from problems in how insulin is produced, how insulin works, or both. Diabetics may develop serious complications such as heart disease, stroke, kidney failure, blindness, and premature death.” The Report pointed out that until 2014, there were 28.9 million of 20 years or older people who had been diagnosed with diabetes, which equaled to approximately 12.3% of the population of the United States. The fact implies that diabetes has emerged as a greatly concerned health issue.

Previous literature has been concentrating on a great number of video-based social media research on YouTube including statistical results of Epilepsy-related resources [9], organ donation [10], retrieval algorithms [11], and quality assessment of patient education content [12]. Diabetes-related research from the perspective of consumers’ health has been conducted as well, such as rural women diabetics [13], Internet-based support groups [14], and diabetes self-management Websites [15]. However, research focusing on diabetes-related video clips on YouTube is still scant in the literature, few studies focus on users’ attitude towards videos on social media, let alone users’ attitude towards health-related videos on YouTube. To fill the gap, this study aims to investigate the factors influencing users’ attitude towards diabetes-related videos on YouTube.

The findings of this study would benefit contributors, users, and designers of a video-based social media like YouTube from three aspects: (1) understanding the characteristics of the diabetes-related user-generated content on YouTube; (2) discovering content themes among diabetes-related video clips on YouTube; and (3) providing useful suggestions to video creators, who are also named YouTubers, and information seekers of diabetes-related information based on users’ preference.

## Method

### Research problem and research questions

Users’ attitude has two sides: positive and negative. This study explores the influencing factors for both positive and negative sides of users’ attitude. Five research questions are stated: (1) what are the characteristics of diabetes-related information on YouTube? (2) What are the factors influencing users’ positive attitude towards the diabetes-related videos on YouTube? (3) What are the associations between these factors and users’ positive attitude? (4) What are the factors influencing users’ negative attitude towards the diabetes-related videos on YouTube? (5) What are the associations between these factors and users’ negative attitude? In the research questions, characteristics mean the specific features of the collection of the investigated diabetes-related videos and factors are the attributes of a video that potentially affect the users’ attitude.

### Research method description

A mixed research method takes advantages of both quantitative and qualitative methods to solve a complicated problem in a study. Both coding and statistical methods were applied in this study. The qualitative method was used to reveal hidden characteristics and theme pattern of the investigated YouTube videos while the quantitative method was employed to investigate the factors influencing users’ attitude towards those videos.

### Selection of a multimedia-based social media website and data collection

In this study YouTube was selected as a data source because it is one of the largest and most popular video sharing platforms in the world [16]. The video clips on YouTube contain a wide range of subjects, such as techniques, sports, health, fashion, music, and so on. YouTube provides several ways for users to seek videos, including subject browse, query search, and personalized recommendation. The users can also subscribe YouTube channels in order to received notifications of new videos created in those channels. A search box on YouTube allows the users to search videos by queries; to filter retrieved results by upload date, type, duration, and feature of videos; and to sort results by upload date, view count, or users’ rating results.

The researchers used diabetes-related search terms to retrieve relevant video clips. The search terms were diabetes, diabetic, type 1 DM (diabetes mellitus), and type 2 DM. For each search term, the videos on top 50 pages were examined. Noticed that there were dead links and private videos that the researchers could not access. In addition, there were duplicate videos returned on different pages or retrieved by different search terms. The duplicate and inaccessible ones were excluded from the raw data set.

### Identification of meaningful attributes or factors of a video clip

After the videos on YouTube were retrieved, each video and its related information were manually examined and the irrelevant videos were excluded by the researchers. A video’s page usually contains a video clip, video clip’s title, the number of views, the number of likes, the number of dislikes, video creator, publish time, a brief introduction of the video, the number of subscriptions, comments of the video, and some recommended videos in the “Up next” section.

The information of the relevant videos was collected, but not every relevant video met the requirements for data analysis. Users’ rate information (the number of likes and the number of dislikes) of a video represents users’ attitude towards the video. The number of likes reflects users’ positive attitude while the number of dislikes shows their negative attitude. Since this study aims to explore users’ attitude, the videos without users’ rate information were excluded.

In the attribute extraction process, useful attributes were identified and extracted from the investigated videos. The attributes included: the number of views, post period, video duration, presenter’s gender, presentation setting, presenter role, media type, subjects of a video, and rating data (the number of likes and the number of dislikes). Some of these attributes were found directly from the Web pages of the videos, such as the number of views, post period, video duration, and rating data (the number of likes and the number of dislikes); some were obtained after examining the videos, such as presenter’s gender, presentation setting, presenter role, and media type; and subjects of a video were identified and assigned after the researchers conducted content analysis for the videos.

The number of views is a unique attribute which has a close connection with the number of likes or dislikes because a user can like or dislike a video only after s/he views it. Both the number of likes and the number of dislikes reflect users’ attitude to a YouTube video. The remaining attributes (the number of views, post period, video duration, presenter’s gender, presentation setting, presenter role, media type, and subjects of a video) were the possible factors influencing the users’ attitude.

### Subject analysis

Subject analysis can give an overall picture of the content coverage of the diabetes-related videos on YouTube. The original subject categories of the relevant videos came from a schema in Zhang and Zhao’s paper [17]. It was used as initial subject categories based on which new subject categories were developed. Table 1 displays the original subject categories from the Zhang and Zhao’s paper.

**Table 1:** Subject categories of diabetes-related content [17]

No.	Category
1	Cause & pathophysiology
2	Sign & Symptom
3	Diagnosis & Test
4	Organ & Body Part
5	Complication & Related Disease
6	Medication
7	Treatment
8	Education & Info Resource
9	Affect
10	Social & Culture
11	Lifestyle
12	Nutrient

After the titles, descriptions, and transcripts of the videos and the videos themselves were examined, subject analysis was conducted. The videos related to the subjects in Table 1 were grouped into the related categories, respectively. The videos which were not related to any subject on the original schema were labeled by the researchers and then the labels were compared with each other. The labels with more similarities were grouped together to generate new categories. The original subject categories which had no connections with the investigated videos were removed. Then the new subject categories were generated. In this study, each video could have one or more subjects. For instance, the video *Living with diabetes – a mum and son’s story* was related to both medication and treatment of diabetes. In other words, a video could be assigned to more than one subject category.

### Regression modeling

Regression modeling analysis helps the researchers explore the identified factors’ influence on the users’ attitude because this method can effectively deal with multiple factors on different data types. Negative binomial regression is a generalization of Poisson regression. It can be applied to a study when: (1) the values of the dependent variable are non-negative integers; (2) the data of the dependent variable are over-dispersed; and (3) the independent variables are in different data scales [18, 19].

In this study both the positive attitude and the negative attitude were the dependent variables. The positive attitude was measured by the number of likes of a video and the negative attitude was measured by the number of dislikes of a video. Both the number of likes and the number of dislikes were non-negative integers. Overdispersion was found in the number of likes data and the number of dislikes data obtained, respectively. The mean of the number of likes was 218.029 and the variance was 917,496. The mean of the number of dislikes was 6.636 and the variance was 390.486. Each variance was much larger than the corresponding mean. In this situation, the Poisson regression model was not applicable, while the negative binomial regression model, was utilized to solve the overdispersion problem.

Post period, video duration, presenter’s gender, presentation setting, presenter role, media type, and subjects of a video were the independent variables for each dependent variable. Among these independent variables, post period and video duration were numeric, while the presenter’s gender, presentation setting, presenter role, media type, and subjects of a video were categorical. Each subject was regarded as a two-level categorical independent variable. If a video was related to a specific subject, its corresponding value for the variable was 1; if not, then the value was zero. The independent variables were on different data scales. As a result, the negative binomial regression model could be utilized.

The negative binomial regression model was frequently used in geometric design, medicine, and health science fields [20], but only a few information science studies applied this model. Didegah and Thelwall utilized the zero inflated negative binomial regression to identify factors influencing citation counts and they found that the impact factor of the publishing journal and the citation impact of the cited references were the most effective factors [21].

The model used in this study is:

$$ \mathrm{E}\left({Y}_i\right)=\mathit{\exp}\left(\mathit{\ln}\left({t}_i\right)+{\beta}_0{x}_{0i}+{\beta}_1{x}_{1i}+\dots +{\beta}_k{x}_{ki}\right) $$

*Y* is the dependent variable (positive attitude or negative attitude in this study), *i* represents a specific video and *Y*_*i*_ stands for the number of likes or dislikes of video *i*. *x*_*ji*_ (*j* = 1, 2, …, *k*) is the *j*th independent variable that describes an attribute of video *i*. *β*_*j*_ (*j* = 1, 2, …, *k*) represents the coefficient of the *j*th independent variable (*x*_*ji*_) of video *i*. *x*_0*i*_ equals to one for all *i* and *β*_0_ is the intercept. *t*_*i*_ is an exposure variable that represents the number of times that the events happen. In this study, the number of times was measured by the number of views of a video. Therefore, the exposure variable is the number of views of a video. In this model exp() is the natural exponential function and exp(*x*) = *e*^*x*^. The significance level for the regression tests was set to 0.05. It was employed as a criterion for hypothesis testing. Table 2 displays the details of the exposure variable, independent variables, and dependent variables defined in this study.

**Table 2:** Details of the identified variables

Variable	Definition	Variable type	Data type
Number of views	Number of views of a video	Exposure	Numeric
Positive attitude	Number of likes of a video	Dependent	Numeric
Negative attitude	Number of dislikes of a video	Dependent	Numeric
Post period	Number of days a video exists since the video was created	Independent	Numeric
Video duration	Length of a video clip measured by seconds	Independent	Numeric
Presenter’s gender	Gender of presenters in a video clip (e.g. female, male, etc.)	Independent	Categorical
Presentation setting	The place where the presentation in a video clip takes place (e.g. office, home, natural setting, etc.)	Independent	Categorical
Presenter role	Presenters’ role in a video clip (e.g. reporter, medical professional, parent, etc.)	Independent	Categorical
Media type	Types of presentation in terms of its source (e.g. TV news, public service announcement, user-generated video, etc.)	Independent	Categorical
Subject_1/2/…/n_	Subjects of a video clip in terms of its content	Independent	Categorical

## Results

### Descriptive results

After examining all the YouTube videos retrieved, we obtained 206 qualified diabetes-related videos. Table 3 presents the levels of the categorical variables identified in this study, which are the presenter’s gender, presentation setting, presenter role, and media type.

**Table 3:** Levels of the categorical variables

Variable	Levels
Presenter’s gender	Male, female, both, not identified, and none
Presentation setting	Home, clinic, studio, outdoor, and others
Presenter role	Reporter, journalist, medical professional, parent, patient, and others
Media type	TV news, TV documentary, interview, user-generated video, public service announcement, and others

**Fig. 1:**
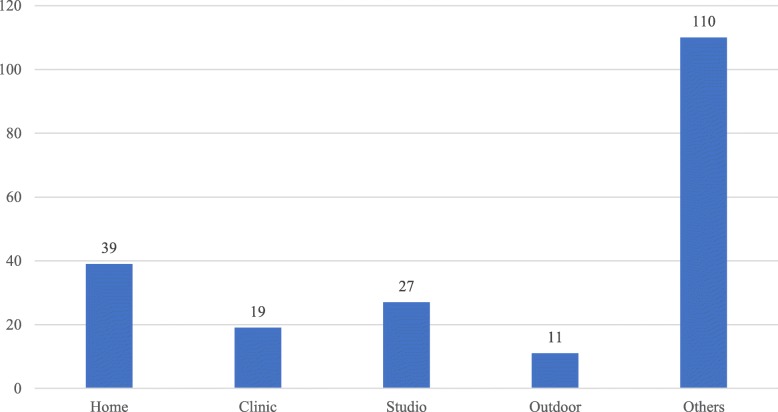
Distribution of settings of the diabetes-related videos on YouTube

**Fig. 2:**
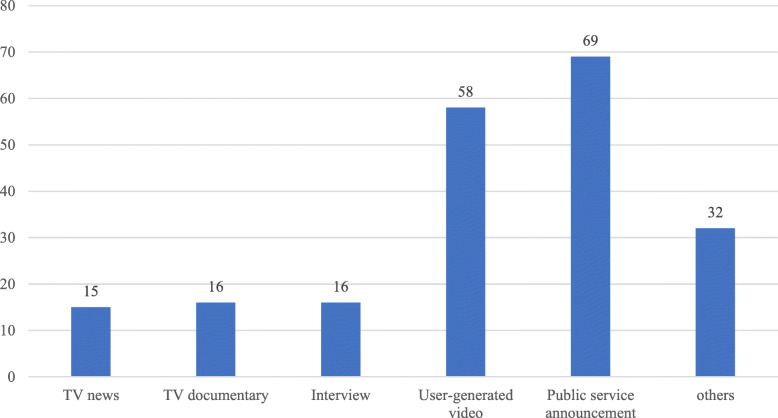
Distribution of media types of the diabetes-related videos on YouTube

Figures 1 and 2 illustrate the distribution of the videos according to the presentation settings and media types, respectively. In the two figures, the *Y*-axes represents the number of videos while the *X*-axes of Fig. 1 refers to the presentation settings. In Fig. 1 the distribution presents that home, clinic, studio, and outdoor place were the main settings of the diabetes-related videos on YouTube. Among these four main settings, home occurred the most (number = 39). The number of videos shot in studios (number = 27) was the second largest comparing to the other three settings. Clinic videos (number = 19) and outdoor videos (number = 11) occupied the third and fourth places respectively. The rest videos were classified into the Others category. This category included the settings like animation scenes, PowerPoint slides, classrooms, and so forth. The large number of the videos in the Others category indicates that the settings of diabetes-related videos were diverse.

The *X*-axis of Fig. 2 stands for the media type. Figure 2 demonstrates that there were 69 public service announcements, more than the other types. User-generated video type was the second largest category, including 58 videos. The videos from TV news (number = 15), TV documentaries (number = 16), and interviews (number = 16) were the least among the six types of video sources. These findings imply that public service announcements and user-generated videos were the primary sources of the diabetes-related videos on YouTube. The finding reveals that individual users contributed relatively less to diabetes-related videos than organizations and agencies.

**Fig. 3:**
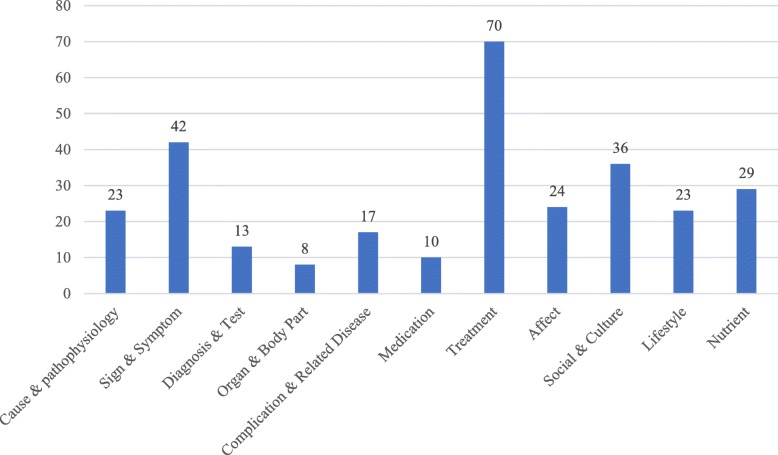
Distribution of video subjects

The results of the subject analysis show that there were 11 subject categories extracted from the 206 videos, which were Cause & Pathophysiology, Sign & Symptom, Diagnosis & Test, Organ & Body Part, Complication & Related Disease, Medication, Treatment, Affect, Social & Culture, Lifestyle, and Nutrition. Compared with the original subject categories, no new category was discovered in this study, while one original subject category, Education and Info Resource, was removed because no relevant video was retrieved in this category. Table 4 lists the codes and the corresponding subject categories of the investigated diabetes-related videos. Since a video might have more than one subject, it could be assigned to multiple subject categories. The distribution of the 11 subject categories is shown in Fig. 3.

**Table 4:** Subjects of the retrieved videos

Code	Subject category
Subject_1_	Cause & pathophysiology
Subject_2_	Sign & Symptom
Subject_3_	Diagnosis & Test
Subject_4_	Organ & Body Part
Subject_5_	Complication & Related Disease
Subject_6_	Medication
Subject_7_	Treatment
Subject_8_	Affect
Subject_9_	Social & Culture
Subject_10_	Lifestyle
Subject_11_	Nutrient

Figure 3 reveals that the Treatment category, which contained 70 videos, reached the first position among the 11 categories. The Sign & Symptom (the number of videos = 42), the Social & Culture (the number of videos =36), and the Nutrient (the number of videos =29) categories received the second, third, and fourth places, respectively. The Affect (the number of videos =24), the Cause & Pathophysiology (the number of videos =23), and the Lifestyle (the number of videos =23) categories were smaller than the previous ones, but larger than the rest categories. The last four places were clenched by the Complication & Related Disease (the number of videos =17), the Diagnosis & Test (the number of videos =13), the Medication (the number of videos =10), and the Organ & Body Part (the number of videos =8) categories, respectively. These results indicate that the subjects of diabetes-related clips on YouTube were various and covering different areas. Treatments and symptoms of diabetes were popular topics on YouTube.

### Inferential statistical results

The factors’ influences on the positive attitude and the negative attitude to diabetes-related video clips were examined by a series of negative binomial regression tests. The positive attitude was measured by the number of likes and the negative attitude was measured by the number of dislikes. The factors included post period, video duration, presenter’s gender, presentation setting, presenter role, media type, Cause & pathophysiology (Subject_1_), Sign & Symptom (Subject_2_), Diagnosis & Test (Subject_3_), Organ & Body Part (Subject_4_), Complication & Related Disease (Subject_5_), Medication (Subject_6_), Treatment (Subject_7_), Affect (Subject_8_), Social & Culture (Subject_9_), Lifestyle (Subject_10_), and Nutrient (Subject_11_).

For each categorical factor, except the subject factors, the level with the most items among the levels was selected as the base level in the regression testing. If the level with the most items was “others”, then the level with the second most items was regarded as the base level. Because the “others” level of each factor contained various videos that could not be assigned to other levels, therefore it is meaningless to compare any level with the “others” level. Consequently, the “other” level could not be the base level. In this study, the Public service announcement level of the media type factor, the Home level of the presentation setting factor, the Medical professional level of the presenter role factor, and the Female level of the presenters’ gender factor were selected as the base levels, respectively. For each subject factor (e.g. Cause & pathophysiology), zero was the base level. Zero represented that a video was not related to a specific subject, while one represented that it was relevant to the subject.

Tables 5, 6, 7, 8 and 9 display the results of regression testing. In each table, the first column lists the independent variables of the regression model, and the second to fifth columns show the coefficient, standard error, z-score, and *P*-value for each independent variable, respectively. The standard errors measure the accuracy of the coefficients. Together with the coefficients, they were used to calculate the corresponding Z-values and *P*-values. If the P-value is smaller than 0.05, then the corresponding variable has significant influence on the dependent variable. From Tables 5, 6, 7, 8 and 9, the *P*-values smaller than 0.05 are marked with “*”. If the rows of the base levels had no value, then the corresponding cells were filled with hyphen symbols.

**Table 5:** All factors’ influences on the positive attitude

	Coefficient	Standard error	Z-value	*P*-value
Intercept	−3.475	0.362	−9.611	0.000*
Media type (TV news)	−0.055	0.328	−0.168	0.867
Media type (TV documentary)	−0.276	0.285	−0.969	0.333
Media type (Interview)	0.072	0.300	0.241	0.810
Media type (User-generated video)	−0.029	0.225	−0.131	0.896
Media type (Public service announcement)	–	–	–	–
Media type (Others)	−0.653	0.208	−3.141	0.002*
Presentation setting (Home)	–	–	–	–
Presentation setting (Clinic)	−0.093	0.335	−0.278	0.781
Presentation setting (Studio)	−0.519	0.314	−1.651	0.099
Presentation setting (Outdoor)	−0.944	0.363	−2.601	0.009*
Presentation setting (Others)	−0.339	0.258	−1.313	0.189
Post period	−0.001	0.000	−10.751	0.000*
Video duration	0.000	0.000	1.140	0.254
Presenter role (Reporter)	0.080	0.220	0.363	0.717
Presenter role (Journalist)	1.141	0.420	2.717	0.007*
Presenter role (Medical professional)	–	–	–	–
Presenter role (Parent)	0.395	0.464	0.851	0.395
Presenter role (Patient)	0.691	0.244	2.829	0.005*
Presenter role (Others)	0.770	0.219	3.509	0.000*
Presenters’ gender (Male)	−0.877	0.166	−5.285	0.000*
Presenters’ gender (Female)	–	–	–	–
Presenters’ gender (Both)	0.110	0.279	0.394	0.694
Presenters’ gender (Not identified)	−2.039	1.079	−1.889	0.059
Presenters’ gender (None)	0.227	0.294	0.774	0.439
Cause & pathophysiology	−0.108	0.252	−0.427	0.669
Sign & Symptom	−0.192	0.208	−0.923	0.356
Diagnosis & Test	0.353	0.339	1.041	0.298
Organ & Body Part	−0.273	0.382	−0.714	0.475
Complication & Related Disease	−0.449	0.285	−1.577	0.115
Medication	−0.476	0.366	−1.303	0.193
Treatment	−0.303	0.190	−1.600	0.109
Affect	0.025	0.287	0.086	0.932
Social & Culture	−0.299	0.243	−1.228	0.220
Lifestyle	−0.473	0.249	−1.898	0.058
Nutrient	−0.346	0.220	−1.572	0.116

**Table 6:** Four factors’ influences on the positive attitude

	Coefficient	Standard error	Z-value	*P*-value
Intercept	−3.885	0.298	−13.035	0.000*
Media type (TV news)	0.002	0.324	0.005	0.996
Media type (TV documentary)	−0.228	0.276	−0.826	0.409
Media type (Interview)	0.021	0.297	0.070	0.944
Media type (User-generated video)	0.089	0.219	0.408	0.683
Media type (Public service announcement)	–	–	–	–
Media type (Others)	−0.620	0.208	−2.976	0.003*
Presentation setting (Home)	–	–	–	–
Presentation setting (Clinic)	0.127	0.325	0.392	0.695
Presentation setting (Studio)	−0.471	0.314	−1.497	0.134
Presentation setting (Outdoor)	−0.825	0.358	−2.304	0.021*
Presentation setting (Others)	−0.213	0.252	−0.846	0.398
Post period	−0.001	0.000	−11.155	0.000*
Presenter role (Reporter)	0.115	0.216	0.531	0.596
Presenter role (Journalist)	1.081	0.427	2.534	0.011*
Presenter role (Medical professional)	–	–	–	–
Presenter role (Parent)	0.710	0.421	1.686	0.092
Presenter role (Patient)	0.801	0.225	3.557	0.000*
Presenter role (Others)	0.650	0.212	3.066	0.002*
Presenters’ gender (Male)	−0.875	0.159	−5.491	0.000*
Presenters’ gender (Female)	–	–	–	–
Presenters’ gender (Both)	0.092	0.273	0.338	0.736
Presenters’ gender (Not identified)	−1.856	1.053	−1.762	0.078
Presenters’ gender (None)	0.097	0.289	0.337	0.736

**Table 7:** All factors’ influences on the negative attitude

	Coefficient	Standard error	Z-value	*P*-value
Intercept	−7.343	0.485	−15.155	0.000*
Media type (TV news)	0.448	0.451	0.993	0.321
Media type (TV documentary)	0.065	0.404	0.161	0.872
Media type (Interview)	0.670	0.413	1.623	0.105
Media type (User-generated video)	0.309	0.310	0.999	0.318
Media type (Public service announcement)	–	–	–	–
Media type (Others)	−0.168	0.273	−0.617	0.537
Presentation setting (Home)	–	–	–	–
Presentation setting (Clinic)	0.324	0.456	0.711	0.477
Presentation setting (Studio)	0.128	0.419	0.307	0.759
Presentation setting (Outdoor)	−0.817	0.511	−1.600	0.110
Presentation setting (Others)	−0.363	0.363	−0.999	0.318
Post period	−0.001	0.000	−4.470	0.000*
Video duration	0.000	0.000	0.700	0.484
Presenter role (Reporter)	0.075	0.285	0.264	0.792
Presenter role (Journalist)	−1.770	0.817	−2.166	0.030*
Presenter role (Medical professional)	–	–	–	–
Presenter role (Parent)	−0.116	0.569	−0.203	0.839
Presenter role (Patient)	−0.262	0.307	−0.856	0.392
Presenter role (Others)	−0.256	0.292	−0.877	0.380
Presenters’ gender (Male)	−0.038	0.228	−0.168	0.866
Presenters’ gender (Female)				
Presenters’ gender (Both)	−0.309	0.399	−0.773	0.439
Presenters’ gender (Not identified)	−34.740	67,110,000.000	0.000	1.000
Presenters’ gender (None)	0.144	0.416	0.346	0.729
Cause & pathophysiology	−0.082	0.312	−0.261	0.794
Sign & Symptom	−0.892	0.313	−2.846	0.004*
Diagnosis & Test	−0.117	0.535	−0.219	0.827
Organ & Body Part	−0.261	0.474	−0.551	0.582
Complication & Related Disease	−0.680	0.420	−1.620	0.105
Medication	−0.234	0.545	−0.430	0.667
Treatment	−0.366	0.262	−1.396	0.163
Affect	−0.265	0.412	−0.643	0.520
Social & Culture	−0.600	0.348	−1.723	0.085
Lifestyle	−0.377	0.333	−1.132	0.258
Nutrient	0.519	0.278	1.870	0.062

**Table 8:** Five factors’ influences on the negative attitude

	Coefficient	Standard error	Z-value	*P*-value
Intercept	−7.673	0.211	−36.344	0.000*
Post period	−0.001	0.000	−5.106	0.000*
Presenter role (Reporter)	0.281	0.257	1.095	0.274
Presenter role (Journalist)	−1.360	0.720	−1.889	0.059
Presenter role (Medical professional)	–	–	–	–
Presenter role (Parent)	0.069	0.476	0.146	0.884
Presenter role (Patient)	−0.040	0.249	−0.162	0.872
Presenter role (Others)	−0.569	0.269	−2.115	0.034*
Sign & Symptom	−0.759	0.248	−3.056	0.002*
Social & Culture	−0.176	0.264	− 0.668	0.504
Nutrient	0.570	0.259	2.201	0.028*

**Table 9:** Four factors’ influences on the negative attitude

	Coefficient	Standard error	Z-value	*P*-value
Intercept	−7.711	0.202	−38.100	0.000*
Post period	−0.001	0.000	−5.062	0.000*
Presenter role (Reporter)	0.229	0.253	0.905	0.366
Presenter role (Journalist)	−1.412	0.713	−1.980	0.048*
Presenter role (Medical professional)	–	–	–	–
Presenter role (Parent)	0.002	0.476	0.004	0.996
Presenter role (Patient)	−0.040	0.248	−0.160	0.873
Presenter role (Others)	−0.588	0.267	−2.206	0.027*
Sign & symptom	−0.721	0.242	−2.979	0.003*
Nutrient	0.613	0.252	2.435	0.015*

#### Factors affecting users’ positive attitude

The first negative binomial regression test examined the influences of all the factors on the positive attitude. Table 5 shows the results of the regression test for all the factors. The results reveal that the media type, presentation setting, post period, presenter role, and presenters’ gender had significant influences on the users’ positive attitude towards the diabetes-related videos. The video duration and subjects had no significant influence on the users’ positive attitude.

To further test these factors’ influences, the factors which had no significant influence in the first model were removed from the regression model. Therefore, only the influences of the media type, presentation setting, post period, presenter role, and presenters’ gender were retested in the second test. Table 6 displays the results of the second test.

Table 6 illustrates that the media type factor had significant influence on the users’ positive attitude. If a video was not TV news, TV documentary, interview, user-generated video, or public service announcement, then its number of likes was significantly less than a video which was public service announcement (Z-value = − 2.976, *P*-value = 0.003 < 0.05). The number of likes of a video belonging to the other media type was about 53.8% of the number of likes of a video which was public service announcement (coefficient = − 0.620, exp.(− 0.620)= *e*^(−0.620)^ =0.538). The number of likes of the TV news, TV documentary, interview, or user-generated video was not significantly different from the public service announcement. The results show that the YouTube users preferred the diabetes-related TV news, TV documentary, interview, user-generated video, and public service announcement to the other related video clips.

The results demonstrate that the presentation setting factor affected the users’ positive attitude significantly. The diabetes-related videos whose presentation setting was outdoor had significantly less numbers of likes than the videos whose presentation settings was home (Z-value = − 2.304, *P*-value = 0.021 < 0.05). Since exp.(− 0.825) = 0.438, the outdoor setting videos’ numbers of likes were 43.8% of the home setting videos. It means that the YouTube users liked home setting much more than outdoor. The other settings had no significant difference from the Home setting.

The post period also had significant effect on the positive attitude (Z-value = − 11.155, *P*-value = 0.000 < 0.05). Since the coefficient was − 0.001 and exp.(− 0.001) = 0.999, the value of the post period factor increased one unit, then the value of the positive attitude decreased 0.1%. The results mean that the post period of a video extended 1 d, its number of likes decreased 0.1%. Therefore, older diabetes-related videos usually had less numbers of likes than newer ones.

The presenter role affected the users’ positive attitude significantly according to the results. When the presenter of a video was journalist (Z-value = 2.534, *P*-value = 0.011 < 0.05) or patient (Z-value = 3.557, *P*-value = 0.000 < 0.05), the number of likes was significantly larger than a video whose presenter was medical professional. The number of likes of a video whose presenter was journalist was nearly three times of a video whose presenter was medical professional (exp (1.081) = 2.948). The number of likes of a video whose presenter was patient was two times larger than a video whose presenter was medical professional (exp (0.801) = 2.228). However, there was no significant difference between the videos whose presenters were reporters, or parents and the videos whose presenters were medical professionals. The findings illustrate that journalists and patients in the diabetes-related videos received more positive attitude than the other roles.

**Fig. 4:**
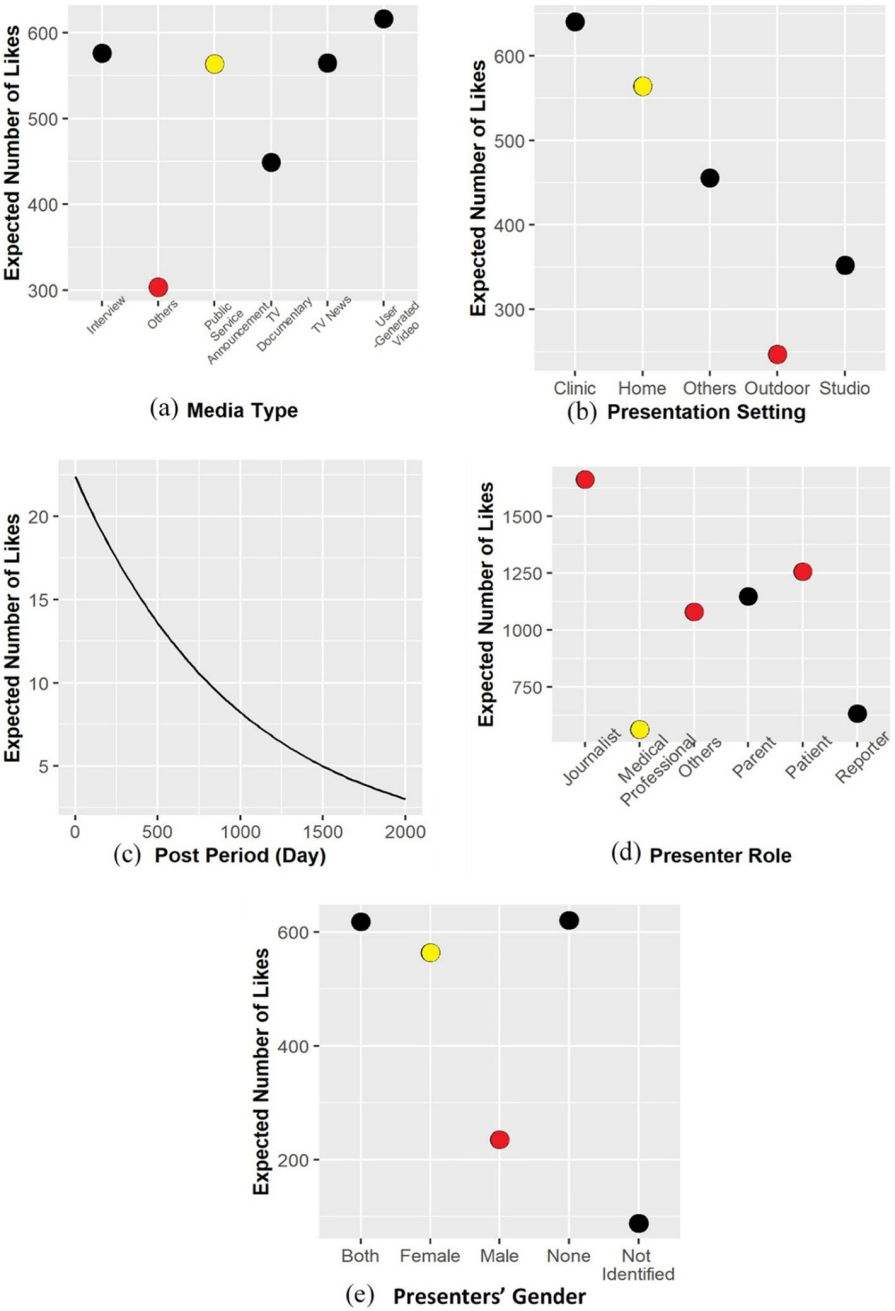
Relationships among factors and users’ positive attitude. Subfigures (**a**) to (**e**) for relationships between (**a**) Media Type, (**b**) Presentation Setting, (**c**) Post Period, (**d**) Presenter Role, and (**e**) Presenters' Gender and number of likes

Regarding the presenters’ gender, the results show that this factor affected the positive attitude significantly. Compared with the videos whose presenters were female, the videos whose presenters were male have significantly lower numbers of likes (Z-value = − 5.491, *P*-value = 0.000 < 0.05). The number of likes of a video whose presenter was male was 41.7% of a video whose presenter was female (exp(− 0.875) = 0.417). There was no significant difference between the videos whose presenters included females, the videos whose presenters’ gender could not be identified, and the videos without human. Therefore, the videos whose presenters were male obtained less positive attitude than the other videos. Figure 4 displays the relationships among the media type (subfigure a), presentation setting (subfigure b), post period (subfigure c), presenter role (subfigure d), presenters’ gender (subfigure e), and users’ positive attitude. Every subfigure in Fig. 4 illustrates the relationship between a factor and the users’ positive attitude. In this figure, the based levels were marked in yellow and the levels that were relatively significant compared with the corresponding base levels were marked in red.

#### Factors affecting users’ negative attitude

The factors’ influences on the users’ negative attitude were tested by the negative binomial regression method. Table 7 demonstrates that the post period, presenter role, and Sign & Symptom subject had significant effects on the users’ negative attitude. The other factors had no significant influence on the users’ negative attitude.

In Table 7, although the subject “Nutrient” and the subject “Social & Culture” had no significant effect on the users’ negative attitude, their corresponding *P* values were close to 0.05 and smaller than 0.1. Therefore, these two factors were included in the second regression model, together with Poster Period, Presenter Role, and Sign & Symptom. The results of the second test are showed in Table 8.

The results in Table 8 reveal that Post Period, Presenter Role, Sign & Symptom, and Nutrient had significant influence on the users’ negative attitude, while Social & Culture did not influence the users’ negative attitude significantly. Therefore, in the third test, only the Post Period, Presenter Role, Sign & Symptom, and Nutrient factors were included as the dependent variables. The results of the third test are displayed in Table 9.

The results in Table 9 demonstrate that the post period had significant effect on the negative attitude (Z-value = −5.062, *P*-value = 0.000 < 0.05). The coefficient is − 0.001 and exp.(− 0.001) = 0.999, so when the value of the post period factor increased one unit, the value of the negative attitude decreased 0.1%. The results mean that the post period of a video extended 1 d, its number of dislikes decreased 0.1%. In other words, older diabetes-related videos usually had less numbers of dislikes than newer ones.

For the presenter role, the results present that this factor had significant effect on the negative attitude. When the presenter of a video was journalist, its number of dislikes was significantly lower than a video whose presenter was medical professional (Z-value = − 1.980, *P*-value = 0.048 < 0.05). The coefficient of the Journalist level was − 1.412 and exp.(− 1.412) = 0.244, which means that the number of dislikes of a video having journalist as its presenter was less than one-fourth of the number of dislikes of a video having medical professional as its presenter. When the presenter of a video was not a reporter, journalist, medical professional, parent, or patient, its number of dislikes was significantly lower than a video whose presenter was medical professional (Z-value = − 2.206, *P*-value = 0.027 < 0.05). The coefficient of the Others level was 0.588 and exp.(− 0.588) = 0.555, which means that the number of dislikes of a video having other role as its presenter was about half of the number of dislikes of a video having medical professional as its presenter. These findings demonstrate that the videos having reporters, medical professionals, parents, and patients as their presenters received more negative feedback than the videos having journalists and other roles as their presenters.

**Fig. 5:**
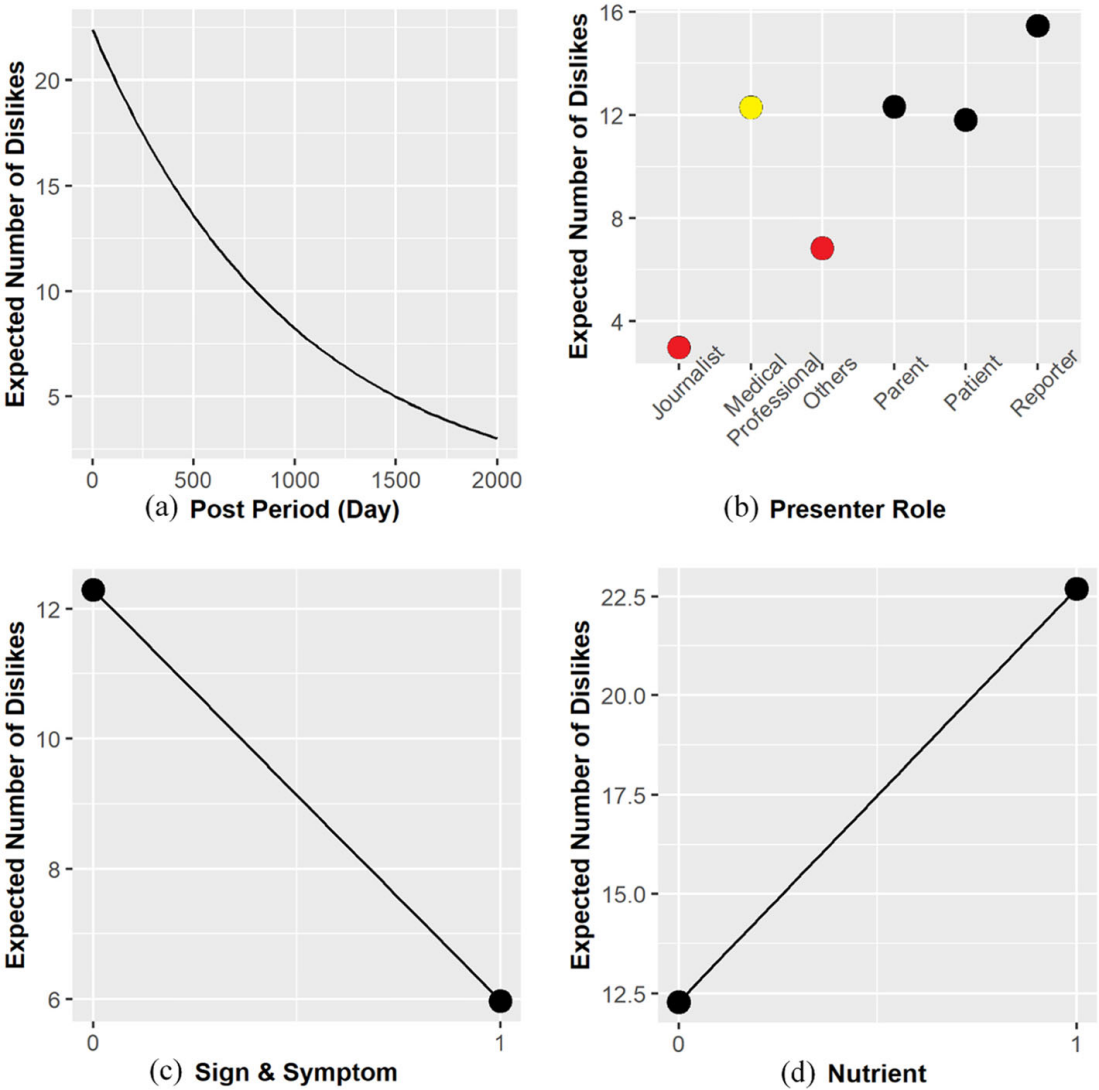
Relationships among factors and users’ negative attitude. Subfigures (**a**) to (**d**) for relationships between (**a**) Post Period, (**b**) Presenter Role, (**c**) Sign & Symptom, and (**d**) Nutrient and number of dislikes

Regarding the subjects, the Sign & Symptom subject and the Nutrient subject both affected the negative attitude significantly. The Sign & Symptom subject had significant negative effect on the negative attitude (Z-value = − 2.979, *P*-value = 0.003 < 0.05). The number of dislikes of a video related to the Sign & Symptom subject was 48.6% of the number of dislikes of a video irrelated to the subject (coefficient = − 0.721, exp.(− 0.721) = 0.486). The Nutrient subject had significant positive effect on the negative attitude (Z-value = 2.435, *P*-value = 0.015 < 0.05). The number of dislikes of a video related to the Nutrient subject was 1.846 times of the number of dislikes of a video irrelated to the subject (coefficient = 0.613, exp. (0.613) = 1.846). Therefore, the users were less likely to have negative attitude on the videos about the signs and symptoms of diabetes, but they were more likely to have negative attitude to the videos about nutrients. Figure 5 displays the relationships among the post period (subfigure a), the presenter role (subfigure b), the Sign & Symptom subject (subfigure c), the Nutrient subject (subfigure d), and the users’ negative attitude. Every subfigure in Fig. 5 illustrates the relationship between a factor and the users’ negative attitude. In this figure, the base level was marked in yellow and the levels that were relatively significant compared with the base level were marked in red.

The findings show that the post period had negative influence on both the positive attitude and the negative attitude. It infers that the users were more likely to show their attitude on new videos than old videos. Meanwhile, the users showed more positive attitude to the diabetes-related videos whose presenters were journalists and patients than the other presenters, and less negative attitude to the diabetes-related videos whose presenters were journalists than reporters, medical professionals, parents, and patients.

## Discussion

One of the distinctive characteristics of social media is users’ participation. Users can not only generate content on a social media Website but also rate items on it. When researchers conduct a study on social media, they can make full use of the user-generated information. For instance, users’ rating data on a social media Website can be employed to evaluate and assess their preference to a certain factor of the social media Website like we have done in this study. The preference rating data are first-hand and natural preference data. They are created directly by real users rather than subjects in an experimental study. Researchers don’t have to conduct an experimental study, or a user opinion survey or interview to obtain the preference information, which has many drawbacks such as time consuming, a lot of extra efforts, a high cost, a relatively small sample size, and inherent bias caused by an experiment or survey or interview. For example, participants may rate preference differently in an experiment or survey or interview because they may be affected by an experiment or survey or interview environment, and tasks may not reflect their real interests/needs.

The results gathered from this study partially echoed with some previous studies. For the characteristics of the video clips on YouTube, the subject categories of “Signs and Symptoms” and “Treatment” had been identified as the majority subjects from both this study and Pant et al.’s research focusing on the acute myocardial infarction [6]. Situations were slightly different in Syed-Abdul et al.’s research where although they found consumers’ favor about the content related to the subject category of “Signs and Symptoms” for Anorexia-related videos, it was also concluded in their article that the videos about subjects “Treatment” and “Prevention” were less favored and had relationship with users’ negative attitude [5]. Regarding the users’ attitudes, this study suggested that compared with medical professionals, diabetes-related video clips made by journalists and patients had significant positive effects on users’ positive attitudes, and the video clips produced by journalists had received significantly less negative feedback. Similar conclusions had been made in Lo et al. [9] and Pant et al.’s [6] articles where the former claimed that “real life” videos made by amateurs generated more hits and comments, as well as the most “favorable empathetic scoring”, than videos created by professionals for epilepsy related content on YouTube; and the latter pointed out that video clips based on personal experience gathered more views and both “likes” and “dislikes” than professionals, pharmaceutical companies, and medical lectures for acute myocardial infarction topic. Moreover, in a review study conducted by Gabarron et al., the authors questioned if video metadata such as the length of a video could be regarded as part of the “Heuristic-Driven Measures” for quality assessment among patient education content on YouTube [12]. They stated that such features should be considered as “heuristics” to investigate a video for the probability of “would be viewed” by consumers instead of substitute for video quality. This study has echoed that claim from the users’ perspective by finding that there was no significant relationship found between video duration and users’ attitudes towards diabetes-related video clips on YouTube.

On the other hand, some findings obtained in this study vary from previous research studies. For instance, the videos such as animation had collected more positive and negative feedbacks than patient experience, news report and professional education for acute myocardial infarction related content [6] while the media type had no significant effect on diabetes audience’s negative attituded in this study. According to Syed-Abdul et al.’s article, the videos with actual body images were more visually appealing to users than the videos without body images in the category of “Treatment” and “Prevention” [5]. In other words, users preferred videos in the “Organ and Body Part” category. However, the phenomenon was not substantiated in this study as there was no significant relationship between users’ attitude and the subject “Organ and Body Part” for the diabetes-related content.

While previous studies mostly focused on the reactions from the users’ perspective to social media content such as the number of views, the number of likes, the number of dislikes, and comments, this study had also shed light on the characteristics of the diabetes-related videos. Through the observation towards the distribution of the videos’ media types, video presenters, settings, and subjects, it reveals that the YouTubers who created diabetes-related videos had their own preferences.

The YouTubers tended to use the user-generated content and public service announcement more than other sources. The reason might be that these two types had the best impact on users based on the YouTubers’ experiences. Therefore, they were expected to receive better attention and feedback. Similarly, the YouTubers preferred home settings over all other settings probably due to the sense of coziness that the home scene could bring to users. These two characteristics indicate that the videos made and published on YouTube were designed and expected to fit the most desired need among the audience.

As the findings presented before, the presentation settings did have significant influence on the users’ positive attitude and the users preferred home settings to outdoor. However, contrary to the video makers’ expectation, although the media type did impact the users’ attitude towards the diabetes-related videos significantly, there was no significant difference among the TV news, TV documentary, interview, user-generated video, and public service announcement. These findings demonstrate that the users’ preference was not always consistent with the YouTubers’ recognition. Therefore, exploration of the users’ preferences is necessary for the YouTubers to help them create more attractive videos.

## Conclusion

Common YouTube users would benefit from the results of this study. The findings shed light on the characteristics of the diabetes-related video clips on YouTube, the diabetes subjects revealed from the data coding analysis, and the factors that affected users’ preferences to diabetes-related video clips on YouTube. These findings help common users effectively browse and access the diabetes-related information on the social media Website. Understanding the user-preferred features and characteristics of a video-based social media Website would facilitate interactions between system and users, especially new users.

System designers can take advantage of the findings from the study. The findings of this study can be used to enhance information organization and information retrieval on diabetes-related video-based social media Websites. The key to a successful social media Website is effective and efficient interactions between the system and its users. Understanding of users’ preferences on content, styles, and other factors would assist system designers in developing a more user-friendly and user-favored system. For instance, the subject schema obtained in the study can be used to organize diabetes-related video clips, which would make users’ navigation smoother; when ranking a returned search results list from a search engine, the retrieved items can be ranked by users’ preferences.

Contributors of video clips can also take advantage of this study. The findings can be used to assist people in effectively creating and presenting video clips on diabetes-related video-based social media Websites like YouTube. One of primary aims of posting a video on a social media is that the video can be shared and accessed by as many viewers as possible. If people understand the factors which affect viewers’ preference to a particular topic on a social media, it definitely would improve the effectiveness of their postings. If users want to create and post a diabetes-related video clip, knowing the user-preferred media types, presentation settings, presenter roles, presenter’s gender, and subjects can help them make more attractive and user-oriented videos.

Video-based social media sites are important information platforms for both health professionals to educate and improve health literacy of the public, and health consumers to learn, share, and publish personalized health-related information and experiences. Although previous studies have focused on users’ health information behaviors on social media Websites and relevant influencing factors, the relationships between the factors and users’ feedback/feelings, especially for diabetes-related content, have not been paid much attention. This study filled this gap through investigating diabetes video clips published on YouTube; detecting the factors that impacted users’ preferences on the video clips from both positive and negative sides; and analyzing the relationships between them.

The findings have three parts: (1) the characteristics (e.g. presenter’s gender, presentation setting, presenter role, media type, and subject) of the investigated diabetes-related video clips obtained from YouTube were examined; (2) several factors that would probably impact users’ preferences of both positive and negative sides to these video-clips were identified. The factors included post period, presenters’ genders, roles, presentation settings, and subjects. Various levels were recognized for each of the factors to further explore the relationships between them and users’ feedback; (3) there were significant relationships between some of the factors and the two sides of users’ preferences. For the positive attitude, significant relationships were detected in the post period, media type, presentation setting, presenter role, and presenter’s gender while for the negative attitude, significant relationships were identified in the post period, presenter role, and two subjects (Nutrient and Sign & Symptom).

The analyses were conducted based on the collected records which contained users’ preference information. Records without users’ preference information were excluded in the data analyses. Users who did not make contributions to preference of records definitely had their preference on the viewed clips. Unfortunately, since the preference data were not kept, they were not taken into consideration in this study, which is a limitation of this study. Future research directions include, but are not limited to, keyword clustering analysis which offers insight into the diabetes-related content on YouTube from a quite different perspective, and studies on other health-related topics such as arthritis and asthma on social media.

## Data Availability

The datasets used and/or analysed during the current study are available from the corresponding author on reasonable request.

## Abbreviation

DM: diabetes mellitus
